# Metabolomic fingerprints of clustered preterm and term neonates – a pilot study

**DOI:** 10.3389/fendo.2025.1569355

**Published:** 2025-05-16

**Authors:** Miłosz Lorek, Teresa Joanna Stradomska, Anna Siejka, Janusz Fuchs, Dominika Januś, Aneta Gawlik-Starzyk

**Affiliations:** ^1^ Department of Neonatal and Pediatric Intensive Care, John Paul II Center for Child and Family Health, Sosnowiec, Poland; ^2^ Department of Pediatrics and Pediatric Endocrinology, Medical University of Silesia, Katowice, Poland; ^3^ Laboratory for the Diagnosis of Metabolic Disorders and Steroidogenesis, The Children's Memorial Health Institute, Warsaw, Poland; ^4^ Department of Paediatric and Adolescent Endocrinology, Jagiellonian University Medical College, University Children's Hospital, Krakow, Poland

**Keywords:** neonates, adrenals, steroidogenesis, metabolomics, K-means clustering

## Abstract

**Introduction:**

Adrenal steroidogenesis plays a pivotal role in neonatal adaptation, and advanced steroid profiling offers novel insights into disease risks and personalized management strategies. This study aimed to identify adrenal steroid metabolomic clusters in neonates and to correlate them with clinical outcomes.

**Methods:**

In a prospective observational design (June 2021–July 2022), 50 neonates (12 early preterm, 18 late preterm, and 20 full-term) admitted with respiratory distress underwent continuous 24-hour urine collection via an urinary catheter. Steroid profiles were analyzed by gas chromatography–mass spectrometry. K-means clustering was employed to classify the metabolomic data, which were subsequently correlated with mortality, bronchopulmonary dysplasia (BPD), small for gestational age (SGA), and intraventricular hemorrhage (IVH).

**Results:**

K-means analysis delineated three distinct metabolic clusters. Cluster 1 displayed a profoundly suppressed steroidogenesis (low C19 and C21 excretion, diminished 3β-hydroxysteroid dehydrogenase and 5α-reductase activities), correlating with an increased incidence of BPD, high mortality risk scores, and significant rates of SGA/intrauterine growth restriction. Cluster 2 exhibited adrenal hyperactivation with elevated cortisol/cortisone derivatives, moderately increased C19/C21 metabolites, and partial 3β-HSD deficits, associated with a heightened risk of IVH and mortality. Cluster 3 showed robust steroidogenesis (high C19/C21 excretion and high 3β-HSD/5α-reductase activities), accompanied by the lowest mortality rates and absence of BPD or SGA/IUGR.

**Conclusions:**

Suppressed steroidogenesis increased BPD, SGA, and mortality, while excessive cortisol output in Cluster 2 was associated with a higher risk of IVH. Robust steroidogenesis supported favorable outcomes, highlighting the potential for metabolome-guided interventions.

## Introduction

Steroids play a pivotal role in the regulation of a multitude of physiological processes during fetal development. The synthesis of these steroids is predominantly undertaken by the fetal zone (FZ) and the definitive zone (DZ), which are crucial regions within the developing fetal adrenal cortex. Dehydroepiandrosterone (DHEA), synthesized in the FZ, undergoes 16α-hydroxylation in the fetal liver and is subsequently converted stepwise to estriol within the feto-placental unit. The synthesis of glucocorticoids and mineralocorticoids occurs in the definitive zone and is determined by the activity of 3β-hydroxysteroid dehydrogenase type 2 (HSD3B2), which facilitates the conversion of pregnenolone and 17-hydroxypregnenolone into their active forms (1–3). Furthermore, recent research has identified the involvement of alternative androgen pathways, such as the 11-oxy pathways, in the production of bioactive steroids, thereby contributing to a more complex understanding of adrenal steroidogenesis (4, 5).

The exact physiological and teleological roles of both estrogens and fetal zone steroids (FZS) in fetal development remain unclear. Nonetheless, recent studies indicate that estradiol, along with FZS such as DHEA, may exert neuroprotective effects. Indeed, the fetal brain expresses estrogen receptors and contains aromatase, an enzyme responsible for local estrogen production (6). Meanwhile, glucocorticoids play an essential role in organ maturation during fetal development, as well as in regulating metabolism and the stress response postnatally. In contrast, mineralocorticoids are crucial for maintaining fluid and electrolyte homeostasis (1–3).

The expansion of the fetal zone commences at approximately 8 to 9 weeks post-fertilization, with steroidogenesis in the DZ developing gradually throughout gestation (7). In the initial stages, the fetal adrenal glands predominantly produce androgens, particularly DHEA, due to the limited expression of the enzyme 3β-hydroxysteroid dehydrogenase type 2 (3β-HSD2) (8). The full capacity for glucocorticoid synthesis is reached during the latter stages of the second trimester, while the significance of mineralocorticoids, such as aldosterone, becomes apparent predominantly in the third trimester (1). As birth approaches, the fetal-placental unit is disrupted and adrenal cortical activity is expected to reach a state of functional maturity. In preterm infants ongoing estriol production from fetal zone DHEA would necessitate the involvement of other peripheral tissues beyond the liver, though the specifics of this process remain unknown (6). Following the expected birth date, the fetal zone of the adrenal cortex gradually undergoes involution (9).

Given the aforementioned characteristics, premature neonates are defined by the immaturity of the adrenal glands, which limits their ability to produce steroids at required levels. Concurrently, there is a high demand for steroids during this intensive perinatal development phase, further complicated by critical conditions associated with premature birth (10). This imbalance in steroid production, particularly in cortisol synthesis, is partially managed by mechanisms such as the balance between 11β-HSD1 and 11β-HSD2, which regulate local cortisol availability in tissues (11).

In practice, the neonatal assessment of the adrenal glands is primarily limited to the exclusion of congenital adrenal hyperplasia (CAH) during screening tests. This involves the assessment of 17 OHP in dried blood spots on filter paper, with further confirmation in a short steroid profile in the same blood sample. This newborn screening enables the exclusion of the most common forms of CAH, including 21-hydroxylase deficiency and 11β-hydroxylase deficiency. In some countries, where screening has not been implemented, exclusion is based solely on clinical suspicion (12). In many cases, genetic analysis is essential to determine the precise cause of primary adrenal insufficiency (PAI), which is of significant importance for the long-term management of the condition, including the personalization of therapy (13). It is also important to note that initial screening diagnostics, involving the measurement of 17-hydroxyprogesterone (17-OHP) levels from blood collected on filter paper from the second day of life, is limited by high false positive rates, and low positive predictive values (14). Proper steroid activity is crucial from birth and disturbances in steroidogenesis have been identified in neonatal disorders such as bronchopulmonary dysplasia (BPD), intraventricular hemorrhage (IVH), and small for gestational age (SGA) (15–20).

The recent literature has focused on the assessment of the steroid metabolome in newborns using gas chromatography-mass spectrometry (GC-MS), given the lack of a commercially available reliable immunoassay for measuring typical neonatal steroids (6, 19, 21). The application of steroid metabolomics allows for the phenotyping of individuals at a chemical level and facilitates the development of cluster-specific therapeutic strategies, which may be particularly beneficial for addressing neonatal disorders. This unique steroid metabolome, often referred to as a metabolic fingerprint, provides insight into specific steroid patterns among metabolites involved in steroidogenesis. By linking these patterns to gestational age or clinical conditions, it enables personalized diagnostics and the tailoring of treatments (19, 22).

Identification of various metabolic patterns is possible through data clustering methods in analysis. One of the most common approaches is the k-means algorithm, which involves dividing a dataset into k clusters, where k is a number predetermined by the user. The earliest mentions of this method date back to the 1960s. The algorithm first assigns all data and then points to randomly selected k cluster centroids and repeats this process until the centroids no longer change, indicating convergence (23). K-means clustering has already been used in steroid analysis for classifying subunits of certain conditions, which aims to personalize the therapeutic approach (22, 24, 25).

By using a k-means clustering algorithm we aimed to compare prospectively the identified steroid metabolomes and the clinical phenotypes of newborns hospitalized in the Neonatal Intensive Care Unit (NICU). Furthermore, the research aimed to elucidate the specific conditions that are characteristic of the steroid patterns identified in preterm and term infants.

## Patients and methods

### Patients

The study population comprised a consecutive series of 50 newborns, including 12 early preterm infants (24%; gestational age (GA): 31 - 31.9), 18 late preterm infants (36%; GA: 34 - 36.7) and 20 full-term infants (40%; GA: 38 - 41.6). The infants were admitted to the NICU within the first 48 hours of life between June 2021 and July 2022. On the third day of life, all patients underwent a 24-hour urine collection to analyze individual steroid metabolites. All patients were routinely catheterized with a Foley catheter due to the need to monitor daily diuresis, making the study non-invasive as the urine collected in this way was used for analysis. Comprehensive data on prenatal growth, labor, and medical histories were gathered from the NICU files to correlate these with the steroid profile outcomes.

The study was conducted in accordance with the Helsinki declaration and the ethical standards set forth by the Ethics Committee of Silesian Medical University in Katowice (PCN/CBN/0052/KB1/99/I/21/22/23) and informed written parental consent was obtained. Given the non-invasive nature of the study, no parental objections to participation were encountered. Previous exclusions included antenatal steroid therapy (n=2) and chromosomal aberration (trisomy 21, n=1). The exclusion criteria encompassed congenital adrenal hyperplasia (CAH), which was not observed. Gestational age was verified using the expanded Ballard score and/or obstetrical dating.

In line with the guidelines of the Pradhan Mantri Surakshit Matritva Abhiyan (PMSMA) for classification of high-risk pregnancy (26), a total of 25 pregnancies in our cohort were classified as high-risk. These included two twin gestations, five cases of urinary tract infections, two cases of preeclampsia, two maternal infections (COVID-19 and pyelonephritis), four cases of placental abruption, three unmonitored pregnancies, three cases of uterine rupture or threat thereof, one case of Hashimoto's thyroiditis, one case of hypothyroidism, as well as additional instances of asthma and polycystic ovary syndrome (PCOS). Cesarean deliveries were categorized based on urgency using a three-tier classification system as described by Torloni et al. (27). Among the 50 neonates included in this study, 35 were delivered via cesarean section, of which 16 were elective, 9 were urgent, and 10 were classified as emergency procedures.

Children were admitted to the NICU due to various conditions, including Infantile Respiratory Distress Syndrome (n = 28), congenital pneumonia (n = 4), respiratory failure (n = 8), low birth weight (n = 7), epilepsy (n = 2), and apnea (n = 1). Broncho-Pulmonary Dysplasia (BPD) was diagnosed according to the 2006 National Institute of Health (US) criteria for BPD (28). Intraventricular hemorrhage (IVH) was evaluated using cranial ultrasound and only grades 1 and 2 were observed (29). SGA was defined as a birth weight below 10th percentile for gestational age (30). Intrauterine Growth Restriction (IUGR) was defined based on prenatal ultrasound criteria as estimated fetal weight (EFW) or abdominal circumference (AC) below the 3rd percentile, or meeting at least two out of three conditions: EFW or AC below the 10th percentile, AC or EFW crossing more than two quartiles, and cerebroplacental ratio (CPR) below the 5th percentile or umbilical artery pulsatility index (UA-PI) above the 95th percentile (31). Both patent foramen ovale (PFO) and pulmonary hypertension (PH) were assessed using transthoracic echocardiography (TNE), following the recommendations of the American Society of Echocardiography (32). TNE was performed within 72 hours after birth in extremely preterm infants and within the first week of life in late preterm and full-term neonates, depending on clinical indications. Sepsis was defined as one positive blood culture with non–coagulase negative staphylococci or one positive blood culture with coagulase-negative staphylococci in combination with a C-reactive protein level greater than 10 mg/L within 2 days of blood culture or two positive blood cultures with coagulase-negative staphylococci drawn within 2 days (33). The severity of illness was assessed within the first 12 hours after admission to the NICU using the Score for Neonatal Acute Physiology Extension-II (SNAPPE-II) (34). All of the patients received both enteral and parenteral feeding.

### Urine collection procedure

Urine samples were collected continuously for a 24-hour period, commencing at 7 a.m. on the third day of life and concluding at 7 a.m. the following day. At six-hour intervals, the urine samples were stored at a temperature of 6 degrees Celsius until the conclusion of the collection period. Upon completion, the total volume of urine collected was recorded, and the samples were then preserved at a temperature of -24 degrees Celsius in a freezer until they were transported in a refrigerated container to the laboratory for analysis.

### Laboratory analysis

The steroid profiles in urine were determined using gas chromatography-mass spectrometry (GC-MS) at the Laboratory for the Diagnosis of Metabolism and Steroidogenesis Disorders with several decades of experience in the analysis of steroid metabolome. The analysis encompassed the monitoring of 30 metabolites in both term and preterm infants (**Table 1**). Steroid analysis was conducted using a Hewlett-Packard HP 6890 Series GC System, which was equipped with a Hewlett-Packard 5973 Mass Selective Detector. The separation was conducted on a 12-meter quartz capillary column, namely the HP Ultra 1. The operational parameters were optimized with the objective of achieving the highest possible resolution and peak sharpness for the steroid compounds. The identification of steroid peaks was achieved by comparing the retention times of the analytes with those of standard steroid compounds under identical chromatographic conditions. This comparison ensured accurate identification based on retention time congruence. Quantitative determinations were conducted by comparing the area of each steroid peak to that of an internal standard, stigmasterol. In cases where compound separation was challenging due to matrix complexity, the Selected Ion Monitoring (SIM) method was employed.

**Table 1:** The list of the urinary steroid metabolites analyzed in the study.

Abbreviation		Urinary Steroid Metabolites	Origin of Urinary Steroid
C19	AN	5α-androstane-3α-ol-17-on (androsterone)	DHEA, androstenedione, testosterone
	ET	5β-androstane-3α-ol-17-on (etiocholanolone)	DHEA, androstenedione, testosterone
	11-OHAN	5α-androstane-3α,11β-diol-17-one (11-hydroxy-androsterone)	Cortisol, 11-hydroxyandrostenedione
	11-OHET	5β-androstane-3α,11β-diol-17-one (11-hydroxy-etiocholanolone)	Cortisol, 11-hydroxyandrostenedione
	DHA	5-androstene-3β-ol-17-on (dehydroepiandrosterone)	DHEA-sulfate
	5-AND	5-androstene-3β-diol	DHEA
	16α-OHDHA	5-androstene-3β,16α-diol-17-one	DHEA-sulfate
	An-3-ol	5α-Androstene-3α,17β-diol	DHEA
C21	5-PT	5β‐Pregnan‐3α,15β,17‐triol‐20‐one	17-hydroxyprogesterone
	16-OHPN	16α-Hydroxy-4-pregnene-3,20-dione	17-hydroxyprogesterone
	17β-OHPN	5β-pregnane-3α,17α-diol-20-one (17a-OH-pregnanolone)	17-hydroxyprogesterone
	17α-OHPN	5α-pregnane-3α,17α-diol-20-one	17-hydroxyprogesterone
	PT	5β-pregnane-3α,17α,20α-triol (pregnanetriol)	17-hydroxyprogesterone
	PTN	5-pregnene-3β,17α,20α-triol (pregnenetriol-17α)	17-hydroxypregnenolone
	PD	5β-pregnane-3α,20α-diol (pregnanediol)	Progesterone
	THS	5β-pregnane-3α,17α,21-triol-20-one (tetrahydro-11-deoxycortisol)	11-deoxycortisol
	THA	5β-pregnane-3α,21-diol-11,20-dione (tetrahydro-11-dehydro-corticosterone)	Corticosterone
	Allo-THA	5α-pregnane-3α,21-diol-11,20-dione (tetrahydro-11-dehydro-corticosterone)	Corticosterone
	THB	5β-pregnane-3α,11β,21-triol-20-one (TH-corticosteron)	Corticosterone
	Allo-THB	5α-pregnane-3α,11β,21-triol-20-one (allo-TH-corticosteron)	Corticosterone
	THAldo	5β-Pregnane-3α,11β,21-triol-20-one-18-al	Aldosterone
Corticosone and Cortisol	THE	5β-pregnane-3α,17 α,21-triol-11,20-dione	Cortisone
	THF	5β-pregnane-3α,11β,17α,21-tetrol-20-one	Cortisol
	allo-THF	5α-pregnane-3α,11β,17α,21-tetrol-20-one	Cortisol
	α -CTN	5β-pregnane-3α,17α,20α,21-tetrol-11-one (α-cortolone)	Cortisone
	β-CTN	5β-pregnane-3α,17α,20β,21-tetrol-11-one (β-cortolone)	Cortisone
	β-CT	5β-pregnane-3a,11b,17a,20β,21-pentol (β-cortol)	Cortisol
	α-CT	5β-pregnane-3α,11β,17α,20α, 21-pentol (α-cortol)	Cortisol
	E	4-pregnen-17α,21-diol-3,11,20-trione (cortisone)	Cortisone
	F	4-pregnene-11β,17α,21-triol-3,20-dione (cortisol)	Cortisol

### Statistical analysis and metabolomic data

The study applied the concept of a "metabolome fingerprint", which was based on the quantitative data obtained from the analysis of urinary steroids by GC-MS over a 24-hour period (35). Peer Group Normalization (PGN) was employed to standardize metabolite values against appropriate reference groups. The selected demographic features included sex, age, body length and body weight, as each significantly influences metabolic profiles. Initially, correlations between these features and metabolites were calculated using Pearson's correlation coefficient to determine their impact. Statistically significant correlations (p < 0.05) formed the basis for assigning weights to each feature. Age was assigned a weight proportional to its number of significant correlations (7), body length with 12 significant correlations, while body weight, with 17 significant correlations, was given the highest weight. Peer groups were constructed considering sex to account for biological differences. For each participant, distances in the feature space (age, body length and body weight) were computed with the inclusion of the assigned weights. Within each peer group, the 10 closest neighbors of the same sex were selected to form the reference cohort, ensuring biological consistency. Normalization was performed by calculating Z-scores for each metabolite based on the mean and standard deviation within the peer group.

**Figure 1:**
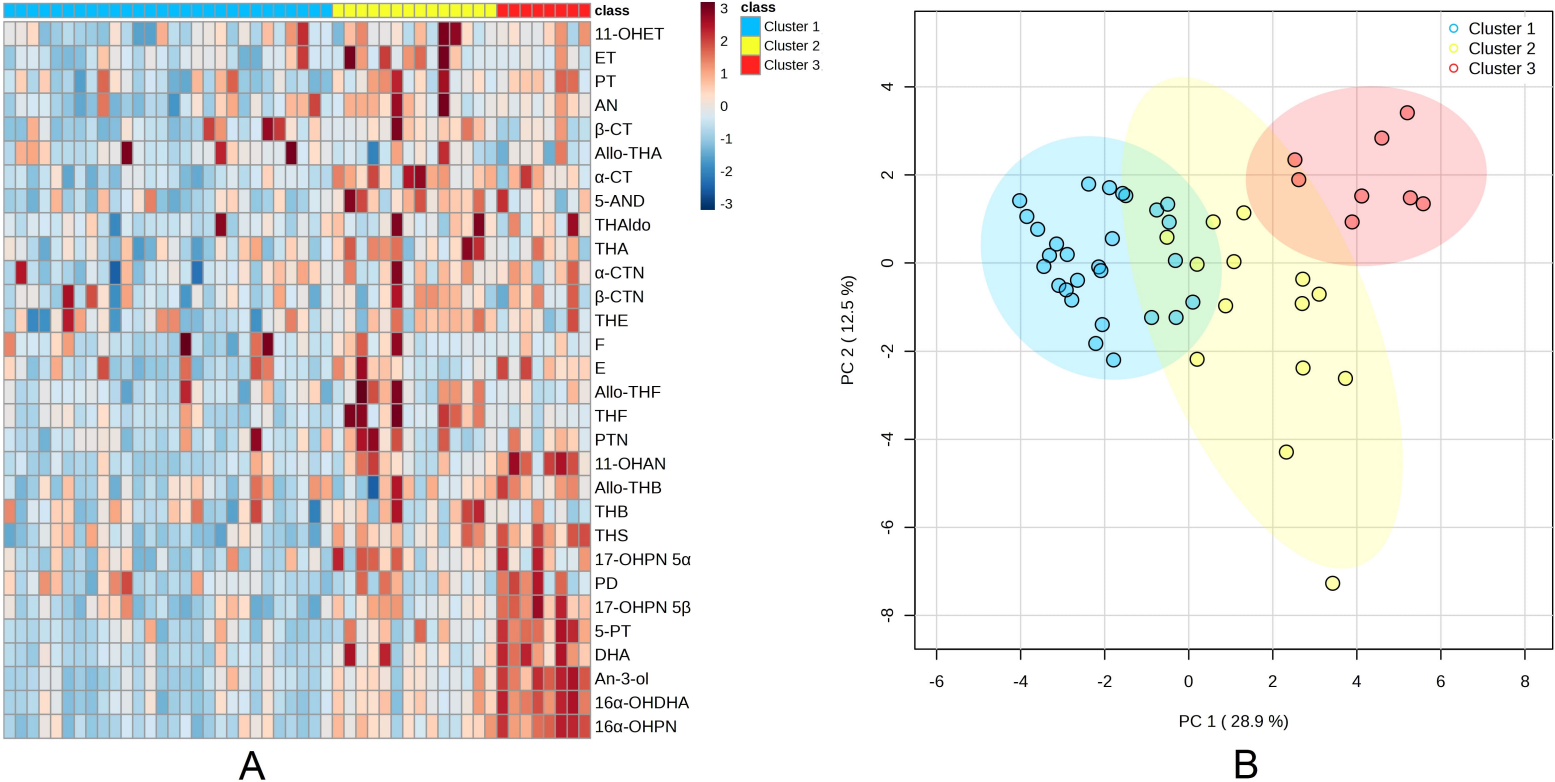
Analysis of steroid metabolite excretion in neonates **(A**) Heatmap and metabolite clusters: The columns correspond to individual patients, while the rows represent the z-transformed 24-hour excretion rates of 30 steroid metabolites. The colour scale employed for the heatmap ranges from blue (indicative of low excretion rates) to red (indicative of high excretion rates). The top marginal row indicates the assigned clusters, **(B)** Principal component (PC) analysis: The dots represent samples from 50 neonates, projected onto the plane defined by the first and second principal components. The colours of the dots correspond to the classification groups of the subjects.

The patient clustering was conducted using MetaboAnalyst, an online platform for metabolomic data analysis (https://metaboanalyst.ca) developed by the Xia Laboratory at McGill University in Montreal, Canada. Three clusters were determined by visual inspection of the heatmap. The k-means clustering algorithm was employed to create three distinct clusters among neonates (**Figure 1A**). The PERMANOVA (Permutational Multivariate Analysis of Variance) and a principal component analysis (PCA) were conducted (**Figure 1B**). Obtained metabolomic fingerprints and clinical data were subjected to statistical analysis using Statistica version 13.0 (StataCorp. LLC, USA), followed by ANOVA and *post-hoc* analysis or Mann Whitney's U Test. The chi-square test or Fisher's exact test was employed to assess the existence of statistically significant clinical differences between the clusters, with a p-value of less than 0.05 considered to indicate such a difference. In order to compare the daily excretion rates of single steroid metabolites between clusters, the Bonferroni adjustment was employed to correct for multiple testing. The metabolites were grouped according to their carbon structure. **Table 1** provides the detailed classification and chemical structures of these metabolites. To calculate the excretion of steroid subgroups, the raw results were aggregated and subsequently, the Z-score was calculated.

### Enzyme activity calculation methods

In the present study, the enzymatic activities involved in steroidogenesis—specifically 11β-HSD-1, 11β-HSD-2, 5α-reductase, and 3β-HSD—were calculated using established ratios between selected precursor and product metabolites, derived from the quantitative GC-MS data. These ratios function as surrogate markers of enzymatic function, thereby enabling inference of pathway activity without the necessity of data normalization. (8, 36).

## Results

**Figure 2:**
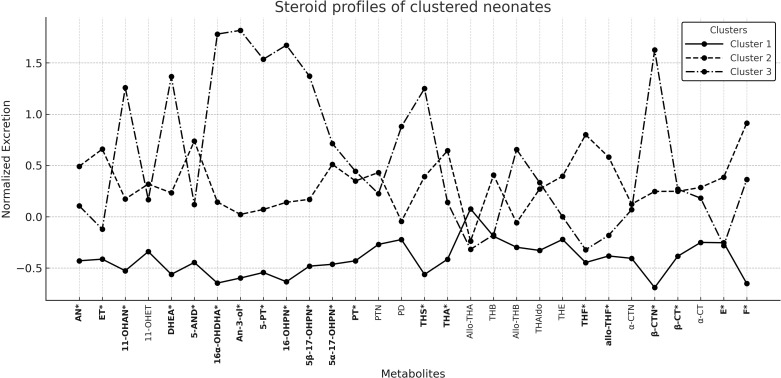
Steroid profiles of Neonate Clusters; *p value < 0.05.

The application of the k-means clustering method resulted in the categorization of all neonates into three distinct clusters: Cluster 1 (n = 28), Cluster 2 (n = 14) and Cluster 3 (n = 8). A subsequent investigation revealed that no statistically significant differences were observed between the neonatal clusters with regard to gestational age (GA), sex or body weight (p > 0.05; **Table 2**). Notably, emergency cesarean sections were significantly more frequent in Cluster 1 compared to Cluster 3 (7 vs. 0 cases, respectively; p = 0.015). **Figure 2** depicts the 'steroid metabolomic signatures' of the three identified clusters. The PERMANOVA analysis demonstrated significant differences between each of the groups in their PCA scores (p = 0.001). The greatest variability among the clusters was related to DHEA and 17-hydroxyprogesterone metabolites. The concentrations of steroid metabolites (raw and normalized data) are presented in **Supplementary Tables 1**, **2**.

**Table 2:** Clinical characteristics of neonatal clusters.

Clinical parameter	Cluster 1 (n=28)	Cluster 2 (n=14)	Cluster 3 (n=8)	P value
Prematurity	20/28	9/14	3/8	0.221
GA	35.0 (29.0 – 41.3)	35.5 (31.0 – 40.9)	37.5 (34.0 – 39.4)	0.652
BW	2350 (1300 – 4075)	2660 (1470 – 3900)	3185 (1950 – 3700)	0.222
Sex (female)	13/28	8/14	4/8	0.804
SGA	9/28	3/14	0/8	0.021^C1 – C3^
IUGR	7/28	1/14	0/8	0.046^C1 – C3^
Apgar 5 min	8 (3 – 10)	8 (1 – 10)	9 (8 – 10)	0.325
High–risk pregnancy	14/28	8/14	3/8	0.683
C-section	20/28	12/14	7/8	0.064
Elective	8/20	7/12	4/7	0.341
Urgent	5/20	3/12	3/7	0.811
Emergency	7/20	2/12	0/7	0.015 ^C1 – C3^
SNAPPE - II	23 (5 – 72)	23.5 (5 – 67)	5 (5 – 24)	0.036^C1 – C3^
LOH [days]	23 (6 – 68)	23.5 (6 – 36)	12.5 (4 – 31)	0.133
IVH	5/28	7/14	3/8	0.033^C1 – C2^
Neonatal Jaundice	11/28	6/14	6/8	0.187
BPD	13/28	5/14	0/8	0.024^C1 – C3^
PPV	20/28	7/14	6/8	0.324
Shock	3/28	1/14	0/8	0.609
EOS	3/28	2/14	0/3	0.551
RBC Transfusion	8/28	5/14	1/8	0.503
PFO	24/28	10/14	7/8	0.498
PH	2/28	1/14	0/8	0.738

GA, Gestational Age; BW, Birth Weight; SGA, Small for Gestational Age; IUGR, Intrauterine Growth Restriction; SNAPPE II, Simplified Newborn Illness Severity and Mortality Risk Score with Perinatal Extension-II; LOH, Length of Hospitalization; IVH, Intraventricular Hemorrhage; BPD, Bronchopulmonary Dysplasia; PPV, Positive Pressure Ventilation; EOS, Early-Onset Sepsis; RBC transfusion, Red Blood Cells transfusion; PFO, Persistent Foramen Ovale; PH, Pulmonary Hypertension.

The *post-hoc* Bonferroni tests demonstrated statistically significant differences between each cluster for C19 and C21 (p < 0.05). A decreased excretion for both C19 and C21 was observed in Cluster 1, an increased excretion in Cluster 3, and moderately elevated excretion in Cluster 2. With regard to cortisol and cortisone derivatives, cluster 2 exhibited high levels of excretion, while cluster 3 demonstrated an intermediate level of excretion; however, cluster 1 presented a significantly decreased result, with a statistical disparity when compared with cluster 2 (p = 0.026; **Table 3**).

**Table 3:** Comparison of steroid metabolite subgroups.

Steroid subgroup	Cluster 1 (n=28)	Cluster 2 (n=14)	Cluster 3 (n=8)	P-value
C-19	-0.812 (-1.366; 0.198)	0.266 (-1.188; 1.058)	1.669 (1.224; 2.474)	0.001
C-21	-0.789 (-1.254; 0.197)	0.333 (-0.497; 1.071)	1.610 (0.898; 2.389)	0.001
Cortisol and Cortisone derivatives	-0.539 (-1.196; 2.806)	0.069 (-0.389; 2.080)	-0.014 (-0.411; 0.950)	0.026^C1-C2^

Data are presented as Median (Min; Max)

The prevalence of SGA was documented in 32.1% of infants in Cluster 1, while IUGR was reported in 25% of pregnancies within this cluster. Notably, no cases of IUGR were observed in Cluster 3 (p=0.021 and p=0.046, respectively). Furthermore, the prevalence of bronchopulmonary dysplasia (BPD) was significantly higher in Cluster 1 (46.4%) compared to Cluster 3 (p = 0.024). Conversely, IVH was observed with the lowest frequency in Cluster 1 (17.8%), exhibiting a substantial difference compared to Cluster 2 (50%; p = 0.033) but not to Cluster 3 (37.5%; p > 0.05). Two early preterm girls from Cluster 3 (25%) exhibited features of androgen excess, evidenced by clitoral enlargement. Cluster 3 was distinguished by the lowest mortality rate, with a median SNAPPE-II Score of 5 (range 5 - 24), exhibiting a significant difference compared to Cluster 1 (23; 5 - 72; p = 0.036) and a trend towards Cluster 2 (23.5; 5 - 67; p = 0.08). The duration of hospitalization was comparable across all groups (p > 0.05).

As demonstrated in **Table 4**, a number of statistically significant differences in steroid metabolite ratios were identified among the neonatal clusters. The highest activity of 5α-reductase and 3β-HSD was observed in cluster 3 (p < 0.002), and increased levels of androgen precursors were also identified (p < 0.002) when compared to clusters 1 and 2. Conversely, the activity of 11β-HSD-1 was higher in cluster 2 than in clusters 1 and 3 (p = 0.021 and p = 0.038, respectively). The activity of 11β-HSD-2 was greater in cluster 1 compared to cluster 2 (p = 0.022).

**Table 4:** Significant differences in steroid metabolite ratios.

Ratio*	Enzyme	Cluster 1 (n=28)	Cluster 2 (n=14)	Cluster 3 (n=8)	P-value
(THF + 5αTHF + α-CT + ß-CT) / (THE + α-CTN + ß-CTN)	11β-HSD-1	0.13 (0.04 – 0.62)	0.24 (0.12 – 0.66)	0.15 (0.11 – 0.19)	0.021^C1-C2^ 0.038^C2-C3^
THE / (THF + 5αTHF)	11ß-HSD-2	15.59 (1.20 – 63.02)	6.60 (0.75 – 28.24)	10.34 (5.43 – 19.90)	0.022^C1-C2^
11-OHAN / 11-OHET	5α reductase	1.91 (0.56; 7.47)	2.82 (1.04 – 8.71	7.86 (1.58 – 11.59)	0.001^C1-C3^ 0.001^C2-C3^
5-PT / PT	3ß-HSD	0.49 (0.07 – 2.51)	0.59 (0.27 – 2.17)	1.70 (0.79 – 2.49)	0.001^C1-C3^ 0.002^C2-C3^
5-PT / (5αTHF+THF+THE)		0.04 (0.01 – 0.47)	0.06 (0.02 – 0.22)	0.20 (0.09 – 0.78)	0.001^C1-C3^ 0.001^C2-C3^
(DHEA + 16α-OHDHA + 16-OHPN) / (AN + ET)		320.69 (37.09 – 1257.10)	408.03 (58.60 – 1100.17)	913.74 (661.83 – 1337.98)	0.001^C1-C3^ 0.002^C2-C3^
DHEA / (AN + ET)		1.01 (0.20 – 4.01)	1.64 (0.09 – 4.61)	4.70 (2.91 – 9.70)	0.001^C1-C3^ 0.001^C2-C3^

Data are presented as Median (Min – Max); Significance by Bonferroni Test. *Calculated on the basis of no-PGN steroid metabolite excretions.

## Discussion

This study utilized a steroid metabolomic signature approach in neonates admitted to the NICU, implementing a rigorous 24-hour urine collection via catheterization in newborns and subsequent analysis by GC-MS (21). Although similar analyses have been conducted using adhesive urine bags or diaper extraction methods (19, 37), these less invasive approaches often face difficulties in ensuring a complete 24-hour sample collection. This highlights the necessity for a standardized sampling technique to accurately capture daily fluctuations in steroid excretion, thereby ensuring the reliability and validity of the metabolomic data.

K-means clustering successfully distinguished three clusters among all neonates that differed in all adrenal steroid synthesis pathways. During gestation, the fetal zone of the adrenal glands predominantly secretes C19 steroid precursors, notably DHEA and its hydroxylated derivatives (9, 38). Consequently, in our dataset, the greatest variation among clusters centered on these C19 metabolites, such as 16α-hydroxy-DHEA and An-3-ol, likely mirroring differences in the pace of fetal zone involution immediately after birth (6). The observed variability in C19 intermediates may reflect distinct adaptive trajectories among neonates, given the established role of the fetal zone in shaping the early postnatal endocrine milieu (9, 38).

In the context of the present study, it was observed that neonates in Cluster 1 exhibited a marked reduction across all three steroid groups, indicating a profoundly diminished adrenal output. In particular, they showed low 3β-HSD activity, as evidenced by minimal downstream products from DHEA (C19) and pregnenolone (C21). Concurrently, diminished 5α-reductase activity curtailed the conversion of androgenic precursors (e.g., testosterone) and the 5α-reduction of cortisol to tetrahydrocortisol. Furthermore, elevated 11β-HSD2 activity indicated heightened placental inactivation of cortisol (11, 39, 40). Collectively, these enzymatic patterns suggest a global suppression of neonatal steroidogenesis in Cluster 1.

Clinically, neonates in Cluster 1 also exhibited elevated SNAPPE-II scores, indicative of an elevated mortality risk relative to Cluster 3. This finding is consistent with previous studies demonstrating that insufficient glucocorticoid availability impairs the stress response in critically ill neonates (41, 42). Given the low excretion of both cortisol and cortisone derivatives in this group, an inadequate adrenal reserve may underlie their higher vulnerability to severe neonatal complications. Such deficiencies in adrenal steroid output appear to compound the risk associated with other perinatal stressors frequently encountered in the NICU. Furthermore, the clinical profile of Cluster 1 was marked by a significantly higher number of emergency cesarean deliveries compared to Cluster 3, suggesting that neonates in this group may have been exposed to more acute perinatal stress. This difference aligns with the overall impression of a less favorable intrauterine and peripartum environment in Cluster 1.

In addition, a significant incidence of bronchopulmonary dysplasia (BPD) was observed in Cluster 1 neonates, with no cases detected in Cluster 3, suggesting a substantially elevated risk in the former group. This observation is consistent with the notion that endogenous glucocorticoids play a pivotal role in lung maturation and the regulation of pulmonary inflammation (6, 16) (28). Consequently, a suboptimal adrenal reserve in these neonates may be a contributing factor to their predisposition for BPD. This finding is consistent with the hypothesis that low cortisol production in preterm infants is associated with poorer respiratory outcomes, and that adequate postnatal steroid levels may be necessary to mitigate the multifactorial pathogenesis of BPD (6, 16).

The findings highlight the potential benefits of targeted steroid therapy as part of a precision medicine approach. Antenatal corticosteroids (ACS) remain the gold standard for pregnancies at risk of preterm delivery, improving neonatal respiratory function and shaping metabolic profiles (43–45). Nevertheless, an effective and safe postnatal regimen to prevent bronchopulmonary dysplasia (BPD) remains elusive. The use of hydrocortisone therapy has yielded equivocal outcomes, with the timing and dosing of treatment proving to be of critical importance (46–49). Initiating treatment beyond 50 days of life is less effective in reducing BPD incidence (50), whereas high-dose glucocorticoids given too early may raise concerns about neurodevelopment (51).

In this context, we propose that individualized postnatal steroid replacement regimens could be optimized using each neonate's steroid metabolomic fingerprint, moving beyond uniform protocols based solely on gestational age. For neonates in Cluster 1, characterized by globally suppressed steroidogenesis, and markedly reduced 3β-HSD and 5α-reductase activity, early low-dose hydrocortisone may serve as a targeted intervention. This concept is supported by the PREMILOC trial, which demonstrated that early low-dose hydrocortisone reduces the incidence of BPD in extremely preterm infants (46). However, our data suggest that neonates with a steroid metabolomic profile indicative of global adrenal insufficiency may particularly benefit from such therapy, if introduced judiciously.

Conversely, in Cluster 2 neonates—who demonstrate elevated cortisol and cortisone levels and a higher risk of intraventricular hemorrhage—the decision to initiate steroid treatment may need to be delayed or withheld, as exogenous glucocorticoids could exacerbate cerebrovascular instability. In these infants, metabolomic screening could serve as a tool for exclusion, helping to prevent unnecessary or harmful intervention.

In cluster 3, where steroidogenesis appears to be preserved and clinical outcomes are favourable, routine postnatal steroid therapy may not be necessary. However, careful long-term monitoring could be considered, particularly in preterm females with signs suggestive of *in utero* androgen excess, given the potential for future metabolic or reproductive complications.

By integrating steroid metabolomic signatures with clinical stratification, this approach supports a personalized model of postnatal glucocorticoid therapy, optimizing timing, dosage, and indication based on objective biochemical markers, rather than generalized treatment algorithms. Such strategies may ultimately enhance respiratory adaptation, reduce complications, and minimize adverse neurodevelopmental outcomes.

Beyond mortality and respiratory concerns, Cluster 1 demonstrated a significant occurrence of SGA and IUGR, although only a small percentage of pregnancies within this cluster were complicated by preeclampsia. Since more than 60% of SGA cases remain idiopathic (38, 52, 53), an attenuated fetal adrenal function, exacerbated by excessive maternal glucocorticoids, could contribute to restricted *in utero* growth (11, 39, 40). Whilst a number of investigations have reported increased DHEA metabolites in IUGR neonates (20, 52), a recent study by Heckmann et al. (2024) similarly identified SGA infants within the cluster of globally reduced steroid excretion, thus reinforcing the notion that broadly suppressed steroidogenesis may help explain the growth deficits observed in Cluster 1 (19).

In the subsequent analysis, neonates in Cluster 2 demonstrated moderately elevated excretion of both C19 and C21 metabolites, in addition to high excretion F and E derivatives, distinguishing them from both Clusters 1 and 3. Alongside this heightened adrenal output, Cluster 2 exhibited a partial deficit in 3β-HSD activity and the highest incidence of IVH. These findings underscore that enhanced steroid synthesis does not necessarily confer neuroprotection in preterm infants. Indeed, while some data suggest that certain FZ steroids may possess neuroprotective capacity—particularly via estrogenic or progestogenic pathways (54, 55)—human evidence remains inconclusive. Del Valle et al. identified robust DHEA production in the fetal zone in the absence of 3β-HSD2 (1, 56, 57), but did not confirm a protective role for these steroids. In contrast, Heckmann et al. established a correlation between heightened FZ activity and an augmented risk of IVH (19). Moreover, an excess or dysregulation of cortisol secretion has been demonstrated to be associated with hemorrhagic complications in preterm populations, underscoring the tenuous equilibrium between beneficial and deleterious steroid effects (41, 42, 58).

From a mechanistic standpoint, elevated cortisol/cortisone derivatives have the potential to destabilize the fragile germinal matrix vasculature, which is highly susceptible to hemorrhage in very preterm infants (29). In this cluster, the high E and F excretion could be both a driver of cerebrovascular fragility and a response to the systemic stress of emerging IVH. Beyond IVH, neonates in Cluster 2 also faced a relatively high mortality risk, suggesting that hyperactivation of the adrenal axis may have broader clinical repercussions. Thus, the adrenal hyperactivation and imbalanced steroid production distinctive of Cluster 2 may, in practice, be more detrimental than protective, raising questions about the roles of backdoor steroid pathways and 11-oxo androgens in shaping cerebral perfusion and vascular integrity. Further research is warranted to clarify whether modulating these pathways could mitigate the cerebrovascular instability observed in this subgroup of preterm neonates.

Finally, neonates in Cluster 3 exhibited the highest excretion of C19 and C21 metabolites, along with intermediate levels of cortisol/cortisone derivatives. This robust steroidogenesis was sustained by elevated 3β-HSD and 5α-reductase activities, hinting at a vigorous yet relatively coordinated adrenal response. This endocrine profile is consistent with the observed low mortality risk in Cluster 3 infants, with no incidences of BPD or SGA/IUGR, thereby supporting the hypothesis that adequate steroid production promotes healthier neonatal adaptation (6, 16) (48),

With regard to IVH, it still occurred in Cluster 3, indicating that any potential neuroprotective influence of robust fetal zone steroidogenesis – such as the proposed protective effects of estrogens or other neuroactive steroids (54, 55) – may not be sufficiently potent to prevent IVH outright. Nevertheless, the overall clinical outlook for Cluster 3 remained favorable, highlighting that this group's balanced adrenal output helps mitigate many of the major complications seen in other clusters.

Beyond short-term outcomes, the distinct adrenal steroid profiles identified in our study may carry important implications for long-term health trajectories. Preterm neonates in Cluster 1, exhibiting globally reduced adrenal steroidogenesis and diminished 3β-HSD and 5α-reductase activity, may be at risk of impaired stress responses and suboptimal neurodevelopment. Although a prospective cohort of ELBW infants found no direct association between low early cortisol levels and neurodevelopmental impairment (41), theoretical concerns persist that prolonged hypoactivity of the hypothalamic–pituitary–adrenal (HPA) axis during critical periods may adversely affect brain maturation and long-term adaptive capacity.

In contrast, Cluster 2 neonates, characterized by elevated cortisol and cortisone levels, may be subject to endogenous glucocorticoid overexposure. Such hormonal profiles have previously been linked to an increased risk of intraventricular hemorrhage and cerebral palsy (41). Moreover, fetal glucocorticoid excess has been implicated in long-lasting alterations of HPA axis regulation, potentially predisposing to hypertension, insulin resistance, and behavioral disturbances in later life (11).

Lastly, although Cluster 3 infants demonstrated favorable early outcomes and preserved steroidogenesis, signs of androgen excess were noted, particularly among preterm females. Two such infants exhibited mild, non-progressive clitoromegaly, suggestive of *in utero* androgen excess. Elevated androgen levels in preterm infants have been associated with accelerated catch-up growth and increased risk of metabolic syndrome later in life (2). In females, these exposures may further predispose to polycystic ovary syndrome (PCOS) or insulin resistance, underscoring the potential long-term implications of early hyperandrogenism. Thus, Cluster 3 exemplifies how a robust and apparently well-regulated steroid profile may favor short-term clinical stability while still conferring select endocrine risks that merit ongoing surveillance.

*in utero*The principal strength of our study lies in its comprehensive coverage of the entire gestational age range, from early preterm to term, in conjunction with a thorough clinical characterization. A further advantage is the 24-hour urinary collection via Foley catheters and GC–MS analysis, a method that allows for complete metabolite assessment and serves to minimize sampling bias.

This study adopted an observational approach, which constrained the capacity to establish definitive causal relationships between steroid profiles and clinical outcomes. Additionally, while the sample population encompassed a diverse demographic, expanding it in future multicenter studies would enhance statistical reliability. Lastly, although clinical and metabolomic data were meticulously collected and analyzed, further prospective validation in larger cohorts is imperative to ascertain the practical applicability of the identified steroid signatures in neonatal care.

The findings of this study demonstrate the potential of metabolomic signatures to facilitate the stratification of neonates according to their adrenal steroid profiles, thus offering a promising avenue for the delivery of personalized neonatal care. However, in order to establish which steroid patterns correlate with specific conditions or outcomes, the execution of larger-scale studies is indispensable. In addition, the application of advanced computational methods, such as artificial intelligence (AI) and neural network approaches, which extend beyond K-means clustering, may provide further insight into these complex metabolic patterns. By leveraging AI-driven analytics, clinicians could more accurately identify, classify, and predict high-risk neonatal phenotypes, thus enhancing diagnostic precision and ultimately improving patient outcomes (59).

## Conclusions

Metabolomic clustering revealed distinct adrenal steroid profiles associated with neonatal health outcomes, highlighting the complex interplay between steroidogenesis and clinical risks. Profoundly suppressed adrenal activity, characterized by low C19 and C21 steroid excretion and diminished 3β-HSD and 5α-reductase activity, was linked to increased risks of mortality, BPD, and growth restrictions (SGA/IUGR), emphasizing the importance of addressing global deficits in steroidogenesis. Conversely, adrenal hyperactivation, marked by elevated cortisol and cortisone derivatives alongside moderately increased C19 and C21 metabolites and partial 3β-HSD deficits, was associated with a heightened risk of IVH and mortality, suggesting that excessive glucocorticoid output can exacerbate neonatal vascular fragility. The most favorable short-term outcomes were demonstrated by neonates with robust steroidogenesis, as indicated by high C19 and C21 excretion and high 3β-HSD and 5α-reductase activity, who exhibited the lowest mortality rates and absence of BPD or SGA/IUGR. These findings emphasize the potential of metabolomic signatures to guide personalized interventions, improving both immediate and future neonatal outcomes.

### Limitations

The study was conducted in a highly specific clinical population—neonates requiring intensive care and urinary catheterization during the first 24 hours of life—which limits the applicability of results to healthy term infants. Furthermore, the absence of a healthy control group precludes direct comparisons with physiological reference patterns. Finally, although 24-hour urine collection offers high diagnostic value, its feasibility is limited to selected clinical settings. These aspects underscore the pilot nature of the study and provide a foundation for future prospective validation.

## Data Availability

The original contributions presented in the study are included in the article/**Supplementary Material**, further inquiries can be directed to the corresponding author.
