# Supplementary material for: Metabolomic fingerprints of clustered preterm and term neonates – a pilot study
Source: Front Endocrinol (Lausanne). 2025 May 16;16:1569355. doi: 10.3389/fendo.2025.1569355 (PMC12122292; doi:10.3389/fendo.2025.1569355)
Supplement: Supplementary file 1 [file Table1.docx]

| Daily Steroid Metabolite Excretion Rates of Neonates Clusters | | | | |
| --- | --- | --- | --- | --- |
|  | Cluster 1 (n=28) | Cluster 2 (n=14) | Cluster 3 (n=8) | p |
| AN | -0.429 (0.889) | 0.492 (1.048) | 0.106 (0.419) | 0.004 |
| ET | -0.413 (0.675) | 0.659 (1.227) | -0.121 (0.516) | 0.009 |
| 11-OHAN | -0.526 (0.417) | 0.173 (0.748) | 1.258 (1.008) | 0.001 |
| 11-OHET | -0.339 (0.789) | 0.319 (1.133) | 0.166 (0.636) | 0.069 |
| DHEA | -0.561 (0.338) | 0.233 (0.936) | 1.366 (0.791) | 0.001 |
| 5-AND | -0.445 (0.688) | 0.738 (0.922) | 0.120 (0.942) | 0.001 |
| 16α-OHDHA | -0.646 0.458) | 0.144 (0.566) | 1.780 (0.535) | 0.001 |
| An-3-ol | -0.597 (0.458) | 0.023 (0.518) | 1.816 (0.582) | 0.001 |
| 5-PT | -0.543 (0.521) | 0.071 (0.763) | 1.535 (0.643) | 0.001 |
| 16-OHPN | -0.633 (0.471) | 0.141 (0.573) | 1.672 (0.519) | 0.001 |
| 5β-17-OHPN | -0.481 (0.709) | 0.170 (0.589) | 1.370 (0.765) | 0.001 |
| 5α-17-OHPN | -0.463 (0.700) | 0.510 (1.042) | 0.713 (1.170) | 0.002 |
| PT | -0.429 (0.815) | 0.349 (0.928) | 0.443 (0.718) | 0.002 |
| PTN | -0.269 (0.777) | 0.431 (1.220) | 0.225 (0.702) | 0.044 |
| PD | -0.222 (0.806) | -0.044 (0.867) | 0.879 (1.216) | 0.061 |
| THS | -0.562 (0.671) | 0.391 (0.720) | 1.249 (0.710) | 0.001 |
| THA | -0.414 (0.693) | 0.644 (1.076) | 0.140 (0.757) | 0.007 |
| Allo-THA | 0.077 (0.984) | -0.238 (0.912) | -0.317 (0.872) | 0.630 |
| THB | -0.190 (0.905) | 0.406 (1.088) | -0.176 (0.618) | 0.208 |
| Allo-THB | -0.297 (0.747) | -0.058 (1.086) | 0.656 (0.735) | 0.081 |
| THAldo | -0.328 (0.703) | 0.270 (1.102) | 0.333 (1.045) | 0.055 |
| THE | -0.221 (0.991) | 0.396 (0.744) | 0.000 (0.878) | 0.068 |
| THF | -0.446 (0.443) | 0.799 (1.248) | -0.321 (0.234) | 0.001 |
| allo-THF | -0.382 (0.644) | 0.582 (1.146) | -0.181 (0.428) | 0.013 |
| α-CTN | -0.405 (0.824) | 0.123 (1.270) | 0.069 (1.007) | 0.997 |
| β-CTN | -0.690 (0.461) | 0.247 (0.477) | 1.625 (0.539) | 0.001 |
| β-CT | -0.385 (0.820) | 0.248 (0.812) | 0.270 (0.688) | 0.020 |
| α-CT | -0.250 (0.909) | 0.286 0.924) | 0.181 (1.020) | 0.071 |
| E | -0.253 (1.028) | 0.385 (0.810) | -0.281 (0.669) | 0.010 |
| F | -0.651 (0.482) | 0.913 (0.989) | 0.363 (0.958) | 0.001 |
| Data after PGN and presented in means (SD) | | | | |
