# Supplementary material for: Metabolomic fingerprints of clustered preterm and term neonates – a pilot study
Source: Front Endocrinol (Lausanne). 2025 May 16;16:1569355. doi: 10.3389/fendo.2025.1569355 (PMC12122292; doi:10.3389/fendo.2025.1569355)
Supplement: Supplementary file 2 [file Table2.docx]

| Daily Steroid Metabolite Excretion Rates of Neonates Clusters | | | | |
| --- | --- | --- | --- | --- |
|  | Cluster 1 (n=28) | Cluster 2 (n=14) | Cluster 3 (n=8) | p |
| AN | 3.921 (2.884) | 11.321 (18.672) | 7.250 (3.131) | 0.004 |
| ET | 1.439 (0.637) | 15.550 (47.261) | 1.788 (0.416) | 0.009 |
| 11-OHAN | 4.879 (2.855) | 10.786 (7.425) | 22.650 (13.980) | 0.001 |
| 11-OHET | 2.339 (0.940) | 3.264 (2.212) | 2.950 (0.778) | 0.069 |
| DHEA | 6.807 (8.366) | 17.829 (15.275) | 46.088 (24.751) | 0.001 |
| 5-AND | 10.636 (8.739) | 20.700 (11.002) | 18.688 (12.924) | 0.001 |
| 16α-OHDHA | 770.914 (794.712) | 1810.879 (983.005) | 4810.100 (1392.432) | 0.001 |
| An-3-ol | 139.275 (96.973) | 232.607 (94.546) | 607.513 (188.527) | 0.001 |
| 5-PT | 9.800 (11.903) | 18.264 (15.195) | 45.313 21.213) | 0.001 |
| 16-OHPN | 326.239 (258.462) | 696.02 (331.250) | 1788.050 (638.892) | 0.001 |
| 5β-17-OHPN | 10.311 (5.175) | 13.629 (4.618) | 24.225 (9.965) | 0.001 |
| 5α-17-OHPN | 0.575 (0.480) | 0.971 (0.593) | 1.013 (0.485) | 0.002 |
| PT | 15.743 (8.720) | 24.329 (13.242) | 26.688 (5.962) | 0.002 |
| PTN | 5.929 (8.925) | 9.657 (8.375) | 8.913 (4.443) | 0.044 |
| PD | 13.546 (15.357) | 22.543 (26.078) | 44.863 (29.367) | 0.061 |
| THS | 7.800 (3.314) | 11.657 (3.737) | 17.463 (4.604) | 0.001 |
| THA | 17.850 (8.824) | 29.721 (18.881) | 25.913 (12.562) | 0.007 |
| Allo-THA | 20.193 (5.744) | 18.436 (2.350) | 18.438 (4.436) | 0.630 |
| THB | 35.157 (68.404) | 67.679 (95.351) | 34.125 16.799) | 0.208 |
| Allo-THB | 75.182 (29.208) | 88.429 (53.258) | 108.275 (24.189) | 0.081 |
| THAldo | 28.093 (21.829) | 45.257 (45.094) | 44.750 (20.685) | 0.055 |
| THE | 170.632 (113.316) | 218.464 (114.253) | 204.650 (60.284) | 0.068 |
| THF | 10.621 (10.403) | 34.829 (27.809) | 15.438 (5.460) | 0.001 |
| allo-THF | 4.489 (11.406) | 11.550 (14.229) | 5.950 (5.571) | 0.013 |
| α-CTN | 28.961 (11.915) | 36.929 (13.900) | 38.550 (9.581) | 0.997 |
| β-CTN | 63.350 (22.334) | 95.586 (71.889) | 80.350 29.267) | 0.001 |
| β-CT | 8.443 (5.057) | 10.786 (3.870) | 7.913 (2.019) | 0.020 |
| α-CT | 11.511 (4.949) | 24.971 (16.080) | 18.975 (7.592) | 0.071 |
| E | 34.393 (31.982) | 45.093 (26.085) | 72.575 (40.497) | 0.010 |
| F | 49.746 (152.264) | 31.193 (27.515) | 24.625 (13.987) | 0.001 |
| Raw data presented as means (SD) | | | | |
